# In-Cell Nanoelectronics: Opening the Door to Intracellular Electrophysiology

**DOI:** 10.1007/s40820-021-00655-x

**Published:** 2021-05-15

**Authors:** Dongxin Xu, Jingshan Mo, Xi Xie, Ning Hu

**Affiliations:** 1 State Key Laboratory of Optoelectronic Materials and Technologies, Guangdong Province Key Laboratory of Display Material and Technology, School of Electronics and Information Technology, Sun Yat-Sen University, Guangzhou, 510006 People’s Republic of China; 2 The First Affiliated Hospital of Sun Yat-Sen University, Guangzhou, 510080 People’s Republic of China; 3 State Key Laboratory of Transducer Technology, Chinese Academy of Sciences, Shanghai, 200050 People’s Republic of China

**Keywords:** In-cell nanoelectronics, Nano-biointerfaces, Intracellular electrophysiology, Electrogenic cells

## Abstract

Establishing a reliable electrophysiological recording platform is crucial for cardiology and neuroscience research. Noninvasive and label-free planar multitransistors and multielectrode arrays are conducive to perform the large-scale cellular electrical activity recordings, but the signal attenuation limits these extracellular devices to record subthreshold activities. In recent decade, in-cell nanoelectronics have been rapidly developed to open the door to intracellular electrophysiology. With the unique three-dimensional nanotopography and advanced penetration strategies, high-throughput and high-fidelity action potential like signal recordings is expected to be realized. This review summarizes in-cell nanoelectronics from versatile nano-biointerfaces, penetration strategies, active/passive nanodevices, systematically analyses the applications in electrogenic cells and especially evaluates the influence of nanodevices on the high-quality intracellular electrophysiological signals. Further, the opportunities, challenges and broad prospects of in-cell nanoelectronics are prospected, expecting to promote the development of in-cell electrophysiological platforms to meet the demand of theoretical investigation and clinical application.

## Highlights

- The factors affecting in-cell nanoelectronics for electrophysiology recording were discussed from versatile nano-biointerfaces, penetration strategies, active and passive nanodevices.
- The applications of in-cell nanodevices in cardiomyocyte and neuron were further reviewed and evaluated.
- The current challenges of nanodevices for intracellular electrophysiology and their potential applications in biomedical fields were discussed.

## Introduction

Numerous significant physiological activities in the human body, e.g. the mechanical contraction of heart and the excitatory conduction of brain, are closely related to cellular electrophysiology. Electrophysiological studies involve many types of cells [1–10], such as cardiomyocytes, embryonic cells, somatic cells, neoplastic cells, neurons and glia cells. Cardiomyocyte and neuron, as the typical electrogenic cell in electrophysiological studies of heart or brain, respectively, have attracted the attention of many researchers. At present, cardiocerebrovascular disease is still the leading cause of morbidity and mortality worldwide, moreover, heart attack and stroke account for 85% of these incidents [11, 12]. To explore the pathogenesis of cardiocerebrovascular diseases for prevention and treatment, it is highly demanded to establish a reliable platform to monitor and analyse the electrophysiology of cardiomyocytes or neurons at single-cell or cell network level [13–20]. Neuroscience and cardiology aim to understand the functional connections of cell circuits, achieve large-scale cell network mapping and explore the function of the brain or heart and their correlation to physiology and pathology [21]. This benefits the electrophysiological recording platform to accommodate thousands of cells for large-scale parallel investigation.

Over the past decades, the conventional methodologies for electrophysiological recording mainly include living animals- or cell-based techniques. Living animals based techniques such as electrocardiography (ECG), electroencephalography (EEG) and magnetoencephalography (MEG) are noninvasive and suitable for analysing large-scale cell aggregate activities, but they offer coarse views with insufficient resolution to precisely record individual neurons or cardiomyocytes [22–28]. To achieve the single-cell recording, a large amount of cell-based techniques and devices were developed. Planar devices can manage a noninvasive, long-term, label-free, multiplexed extracellular recording of cellular electrophysiology. Initially, microelectrode array (MEA) was first proposed and widely employed to perform the extracellular recording of cardiomyocytes and neurons [29–34]. Microelectrodes manufactured on an insulating substrate receive electrical signals from cells, which are then transmitted to an external amplifier by leads covered with passivation layers for signal processing. For sensitive electrophysiological measurements, low electrode impedance with high signal-to-noise ratio (SNR) is necessary. Using a millimetre-scale MEA is an effective way to reduce the impedance and electrochemical noise for detecting tiny membrane ion fluxes [35, 36]. However, the extremely large electrode areas are limited by the low throughput. To enhance the performance of MEAs, many efforts have focused on increasing the density and number of electrodes. The passive MEA ranging from tens to hundreds of electrodes can successfully achieve the multisite extracellular recording in vitro culture [38, 39, 40, 41], while conventional multitransistor array is simultaneously developed from planar silicon (Si) FETs [42, 43] to silicon nanowire (SiNW) ones [45–46]. However, their spatial resolution is both limited due to the necessary layout of electrode leads. To perform the high spatial resolution recordings, the complementary metal oxide semiconductor (CMOS) integration is introduced to significantly reduce the leads at the electrode layer by sharing the leads, which significantly improves the number of electrodes to thousands. Based on the addressable properties, these active devices achieve both single-cell and cell network recording, enabling to regulate cell accurately and map the cell electrical activity conduction [48–52].

These extracellular devices are manufactured by a great variety of materials and approaches, such as Au, indium tin oxide (ITO), titanium nitride (TiN), Si, carbon nanotube, graphene by bottom-up or top-down strategies [33, 34, 37, 45, 46, 51, 54–57], which are conducive to achieving scalable dimensions, stable coupling interfaces, multiplexed measurements and high-resolution signal recordings. For some applications, extracellular recordings are sufficient and cost/time efficient, allowing for noninvasive, long-term and multiplexed electrophysiological detection. However, extracellular recordings have the weak cell–electrode coupling, leading to low signal quality and distorted signal profile compared with the native transmembrane potential, which are difficult to reveal the key properties and features. Intracellular recording can overcome these limitations, because it is close to the transmembrane potential and exhibits outstanding advantages in exploring the instinct mechanisms of signal changes, mapping the connections of cell circuits, and studying the potential regulation of chemical stimulation. Electrophysiological researches based on living animals can only provide the extracellular coarse information for electrophysiological signals. Currently, intracellular recording is the powerful technique to study the transmembrane potential, which will promote the further development of cardiology and neuroscience. Conventional intracellular sensing and recording methods include patch clamps recording, voltage-sensitive dyes (VSDs) or voltage-sensitive fluorescent proteins (VSFPs; optogenetic reporters). The patch clamp technique is the “gold standard” accurate and effective method for intracellular recordings due to its high-fidelity transmembrane potential, which contains high-resolution details for exploring ion channel properties [59–65]. It gradually developed from conventional patch clamp technology [67–70] to patch clamp integrated systems [72–75] and has been widely used in the biomedical field. However, they cannot perform the study on multiple cells in a network in one well. In addition, the irreversible damage to cells and the complex operation process of patch clamp are not conducive to the long-term and simultaneous recording of multiple cells, which has great limitations on the study of neural networks. On the other hand, VSDs and VSFPs can readily achieve the intracellular recoding with high spatial resolution, but these approaches suffer from low signal-to-noise ratios, phototoxicity, and pharmacological side effects [77–83].

Though intracellular recording techniques possess superiority in many aspects, their development is expected to meet the following goals [85–86]: (1) Minimum invasiveness: The electrodes are required to be smaller than 5 μm to penetrate the cellular membrane in a gentle manner [87]. The smaller electrode dimension could reduce the invasiveness to cells, allowing for a reversible, long-term and chronic recording. (2) High sensitivity: The electrode should possess a high sensitivity and SNR to detect subthreshold potentials with amplitudes in the range of ± 0.5–10 mV and sensitively derive the details of weak signals [88]. (3) Large-scale recording: Multiple cell–electrode interfaces afford both single-cell recording and network-scale extension, which contributes to precise positioning and large-scale control. (4) Stable cell–electrode interface: This requires the cell–electrode coupling, and the properties of the electrode (material, surface, geometry, etc.) remain stable, which not only helps to reduce signal loss, but also lays a solid foundation for long-term and complex mechanism research. It is worth noting that cardiomyocytes and neurons are the general study models in the intracellular recording, while the intracellular recordings of neurons are more difficult due to the sparse distribution and specific chemical synapse connections, so low probability and subthreshold signals of neuronal intracellular recordings are both bigger challenges.

The recent advances of nanodevices have encouraged new concepts to satisfy the above requirements. Nanodevices have prominent advantages in dimension to minimize invasiveness and enhance the throughput, which significantly increases the number and density of recording sites [87, 89, 90, 91]. With the development of micro/nanofabrication technologies, the wide selection of materials and the flexible design of structures provide many options to create the nanodevices. The continuous promotions of nano-biointerfaces and penetration strategies also substantially enhance the intracellular recording performance of nanodevices. In particular, combining intracellular electrodes with extracellular MEA structures and CMOS electronic circuits can not only improve the signal quality, but provide feasible strategies for constructing large-scale high parallel platforms as well [92–94]. At present, nanodevices have been widely used in neuroscience and cardiology for drug screening, disease modelling and preclinical diagnosis [9, 95, 96].

**Fig. 1:**
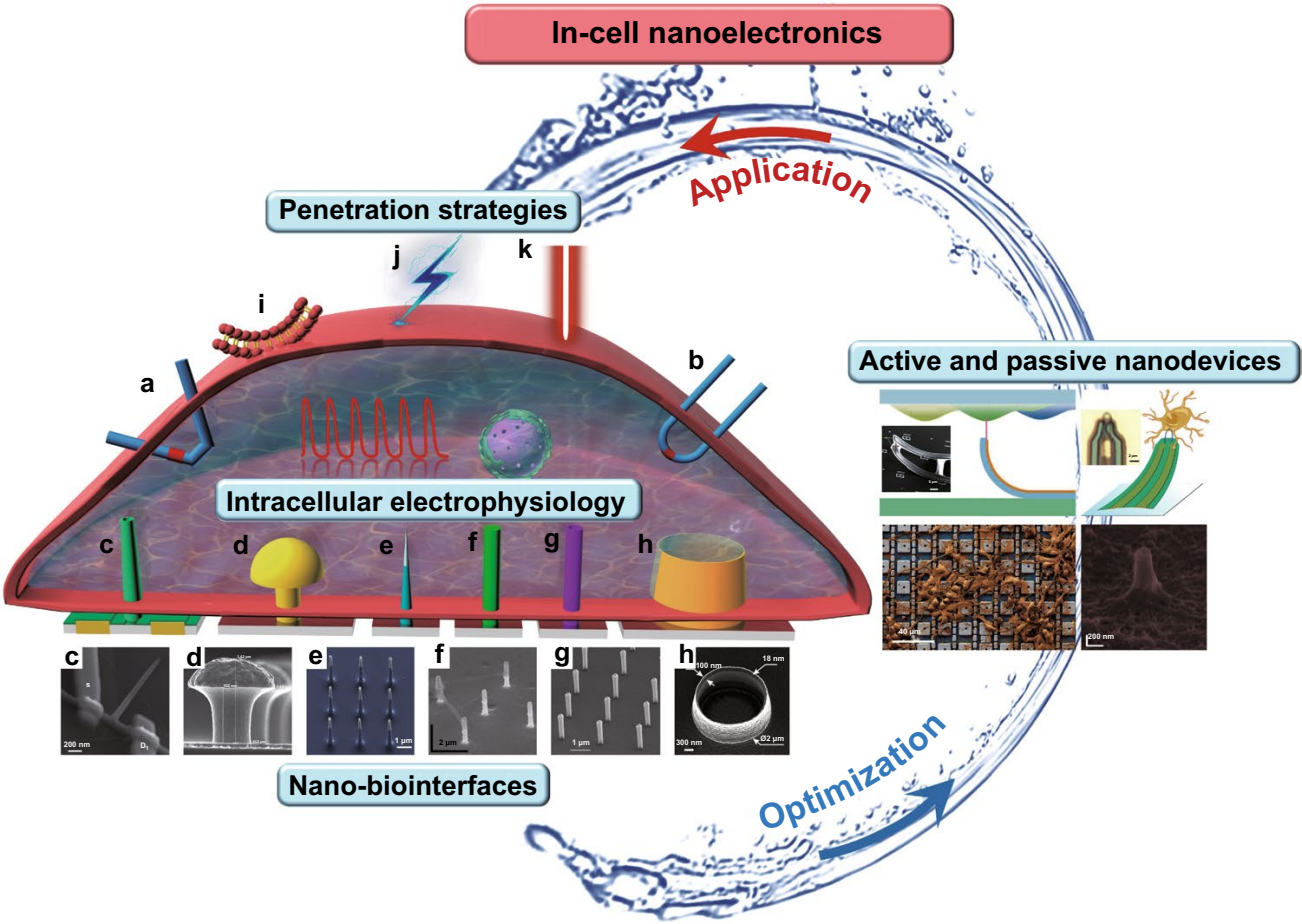
In-cell nanoelectronics for opening the door to intracellular electrophysiology. In-cell nanoelectronics are developed based on the interaction of design optimization and practical application: the continuous optimization of nano-biointerfaces, nanodevices and penetration strategies promotes intracellular electrophysiological detection; the advantages and shortcomings reflected in the application lead to the further development of in-cell nanoelectronics. **a–h** Schematic illustrations and SEM or optical images of different nanodevices, including branched nanotube FET (**a**), gold-mushroom-shaped electrode (**b**), nanowire electrode (**c**), nanopillar electrode (**d**), nanotube electrode (**e**), volcano-shaped electrode (**f**), kinked nanowire FET (**g**), U-shaped nanowire FET (**h**). **i–k** Schematic illustrations of penetration strategies, including chemical modification (**i**), electroporation (**j**), plasmonic optoporation (**k**)

In this review, the new concept of “In-cell nanoelectronics” is proposed as nanoelectronics field for the intracellular investigation and applications. Compared with other bioelectronics, it establishes an intracellular pathway and can reflect the electrophysiological state by high-quality recordings, which is of great significance to study the cellular working mechanism. We describe the recent advances in nanodevices for intracellular recordings (Fig. 1). First, we conduct an in-depth study examining three factors affecting electrophysiology recording: material and structural processing of nano-biointerfaces (Sect. 2), penetration strategies (Sect. 3) and nanodevices (Sect. 4). It is worth noting that the nanodevices are divided into passive and active types, and their recording principle and interface coupling are discussed in detail. Next, we explore the application of nanodevices in cardiomyocytes and neurons by comparing their effects on the high-quality intracellular electrophysiological signals (Sect. 5), demonstrating the superiority and specificity of nanodevices. Finally, we rationally discuss the future development trends of nanodevices and their potential applications in biomedical fields.

## Nanodevice Manufacture

The manufacture of nanodevice involves material selection and structural design, which closely affect the impedance and coupling of the cell–electrode interface. To achieve high-quality signal recordings, numerous attempts have been made to fabricate the nanodevices with less influence of electrode impedance and higher seal of cell–device coupling. Moreover, with the continuous and rapid development of micro/nanofabrication technologies, these nanodevices have been further optimized to achieve high-stability and large-scale detection in high-standard practical applications over the proof of concepts.

### Nanodevice Materials

The choice of device material has an effect on the impedance of a nanoelectrode. At present, nanodevice materials are mainly comprised of biocompatible conductive or semiconductive materials. Conductive materials (e.g. Au and Pt) are commonly used to fabricate the passive metallic electrode, which record the interfacial electric current induced by action potential and electrochemical impedance [10, 91, 95, 97–101]. Meanwhile, semiconductive materials (e.g. Si) are generally applied to fabricate the active transistors, which measured the action potentials on the gate [87, 102–109]. Furthermore, the combination of conductive and semiconductive materials can fabricate nanodevices with silicon cores and metal tips [89], and the functions of nanodevices are further enhanced by CMOS integration [92, 94]. Smaller nanoelectrode will lead to a worse signal-to-noise ratio. However, big electrodes with the diameter of over 10 microns are difficult to perform the intracellular recording, so small electrode with small area is necessary for intracellular recording, which is conducive to perform the intracellular access. Compared to the extracellular signals, the intracellular signals have large amplitude, so the SNR is still increase. To reduce the noise, many strategies are introduced to increase the surface area of the electrode. For example, nanotube structure was fabricated to replace the nanopillar [101]. In addition, electroplating conductive materials, such as Pt-black, carbon nanotubes (CNTs) and conducting polymers [6, 57, 88, 94, 110, 111], can increase the surface area, reduce the electrode impedance and improve the SNR.

### Nanodevice Geometry and Fabrication

**Fig. 2:**
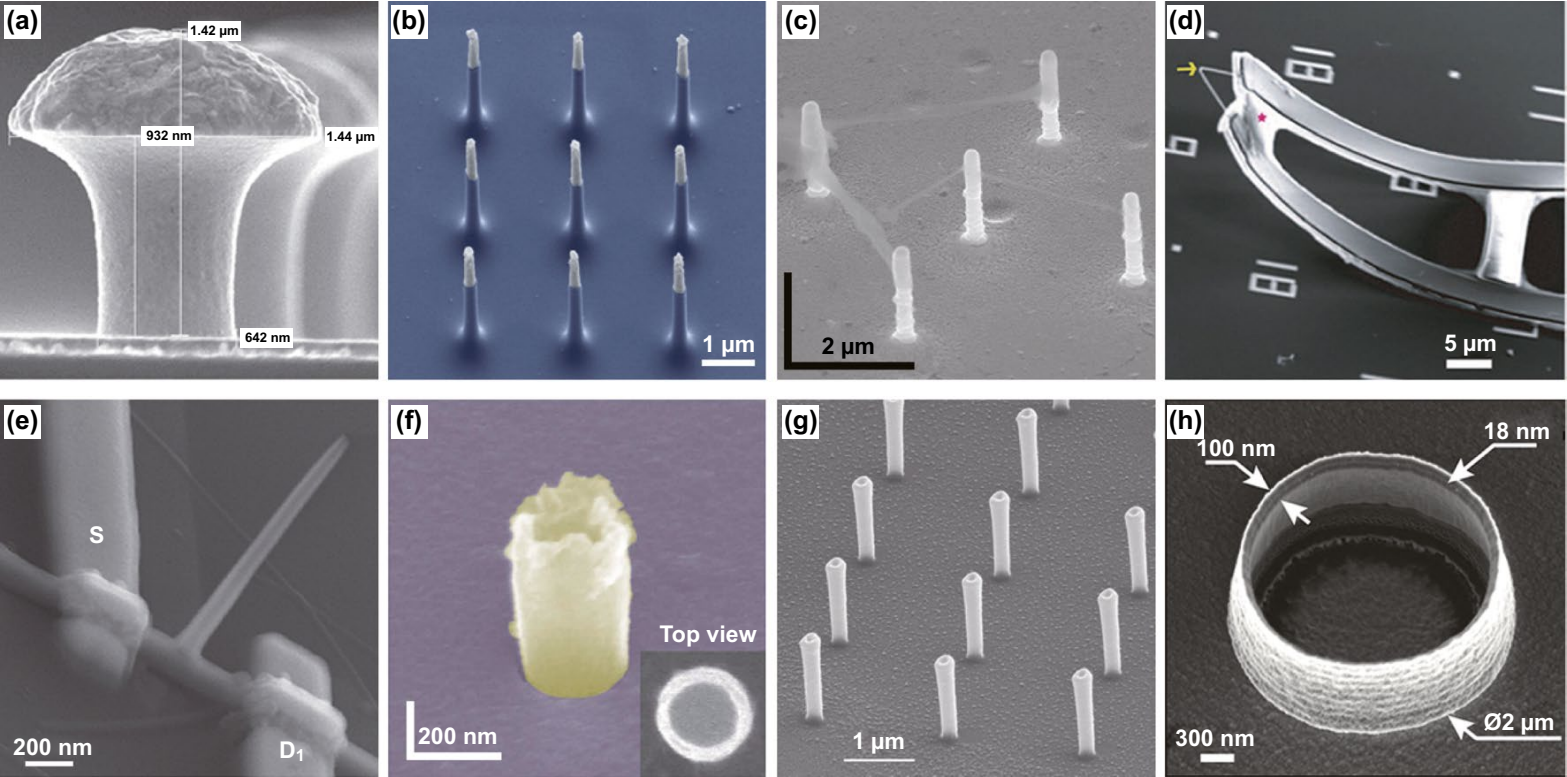
Nanodevices with different materials and geometric shapes. **a** SEM image of the gold-spine electrode. Reproduced with permission from Ref. [98]. Copyright 2010, American Physiological Society. **b** SEM image of the vertical nanowire electrode array with silicon core and gold tip. Reproduced with permission from Ref. [89]. Copyright 2012, Nature. **c** SEM image of the vertical Pt nanopillar electrode array. Reproduced with permission from Ref. [91]. Copyright 2012, Nature. **d** SEM image of the device based on the kinked silicon nanowire. Reproduced with permission from Ref. [87]. Copyright 2010, American Association for the Advancement of Science. **e** SEM image of branched SiO_2_ nanotube field-effect transistor. Reproduced with permission from Ref. [102]. Copyright 2011, Nature. **f** SEM image of the IrOx nanotube electrode. Reproduced with permission from Ref. [101]. Copyright 2014, Nature. **g** SEM image of gold-coated plasmonic 3D nanoelectrodes. Reproduced with permission from Ref. [100]. Copyright 2017, American Chemical Society. **h** SEM image of the nanovolcano electrode with Au nanoring. Reproduced with permission from Ref. [10]. Copyright 2019, American Chemical Society

The design of nanoelectrode geometry plays a crucial role in cell–device coupling. The cleft and loose coupling between the planar nanodevice and cell lower the signal quality. To narrow or eliminate the gap, Hai et al. first designed a three-dimensional (3D) electrode simulating the shape and dimensions of dendritic spines, which were proven to improve cell–electrode coupling (Fig. 2a) [97, 98, 112]. However, the 3D gold-spine electrode possessed a large and round top that was difficult to be engulfed by cells. To enhance the performance of cell penetration, one-dimensional nanomaterials or nanostructures (e.g. nanowires and nanopillar) were integrated for intracellular recording applications (Fig. 2b, c) [9, 90–92, 94, 95, 113]. Compared with the planar or large devices, the 3D sharp nanodevices greatly improve the cell–electrode coupling and facilitate the intracellular access. However, based on passive metallic nanoelectrode paradigm, the seal resistance is too low to derive the full action potentials due to the current leakage. To obtain signal amplitudes comparable to those recorded with patch clamp, active nanotransistor paradigm for cardiomyocytes was introduced to exclude the impact of interfacial impedance (Fig. 2d, e) [87, 102, 103, 107]. Lieber group originally designed a series of kinked nanowire and branched nanotube bioprobes with source/drain (S/D) and nanoscale FET channels. Without the limitation of the interfacial impedance, the dimension of the nanoelectrode can be minimized to achieve a high-density nanoelectrode array. Moreover, nanostructures can significantly affect cell–electrode coupling and intracellular access. Besides, the hollow tubular structure electrode can functionally delay membrane resealing and even induce the active membrane fusion to enhance the seal resistance and prolong the intracellular recordings (Fig. 2f–h) [10, 100, 101]. The study of U-shaped nanowire probes based on the advanced alignment further demonstrates that electrodes with smaller curvature radii facilitate to penetrate the cellular membrane and record full-amplitude intracellular action potentials and subthreshold features in a high-throughput manner [109].

There are two main manufacturing approaches for nanodevices, namely bottom-up and top-down methods [114, 115]. The bottom-up manufacturing strategies are based on the synthesis/assembly of individual atoms and molecules. Among them, the vapour–liquid–solid (VLS) method is popular to synthesize semiconductor nanowires [116–120], which relies on a metal nanoparticle catalyst and promotes the anisotropic growth of semiconductor nanowires in the presence of a liquid–solid interface. Heterogeneous structures can be obtained by adjusting the conditions during the growth process. Other bottom-up synthesis approaches include chemical vapour deposition (CVD) [121, 122], atomic layer deposition (ALD) [123, 124] and metal–organic vapour phase epitaxy (MOVPE) [125, 126]. In contrast, the top-down manufacturing strategies are based on a bulk substrate that is progressively sculpted or etched to obtain predetermined nanostructures. The process usually consists of two steps. First, lithography is carried out under masking with a target pattern, and lithography technology mainly involves photo, colloidal, nanoimprint, electron beam (E-beam), focus ion beam (FIB) or X-ray lithography [127–131]. Next, the material is selectively removed from the substrate, primarily through dry/wet etching, typically reactive ion etching (RIE) and metal-assisted chemical etching (MACE) [132–134]. The top-down manufacturing strategies have outstanding advantages in precisely controlling the structures of nanodevices.

At present, the preparation of most nanodevices combines the above two manufacturing strategies to construct a more flexible and stable structure. For example, in the case of the kinked nanowire probe, a 120° bending structure was obtained by changing the vapour pressure in the VLS reaction, and remote electrical interconnects were made through e-beam lithography to separate the kinked nanowire structure from the substrate (Fig. 2d) [87]. During the preparation of a branched intracellular nanotube FET, the trunk and branched structure was first fabricated by the combination of VLS, electron beam lithography, metal evaporation and ALD, and then etching was performed to finally obtain the nanotube structure (Fig. 2e) [102]. For vertical nanodevices, the target structure was usually constructed by the top-down manufacturing strategies, and the passivation layer (e.g. Al2O3, Si3N4/SiO2) was then deposited by ALD or CVD to electrically isolate the non-active region from the culture medium (Fig. 2b, c) [89, 91]. Recently, great progress has been made in combining nanodevices with industrial CMOS technology. Thousands of nanodevices were fabricated directly on top of a CMOS circuit to achieve large-scale parallel recordings at the network level [92, 94]. Although CMOS fabrication is limited by high-cost and compatible processing technology, it is still an effective strategy to implement high-resolution and high-throughput integration, which has unique advantages in studying cellular network electrical activity. The materials and processes of nanodevices can be unified one by CMOS fabrication. The large-scale parallel fabrication techniques, such as photolithography and electrodeposition, are more conducive to develop high-fidelity and high-throughput nanodevices for satisfying the commercial requirements.

## Intracellular Access Strategies

To obtain a high-fidelity intracellular signal, the probe of nanodevice is demanded to penetrate the cell plasma membrane and form a tight coupling with the membrane. High-performance intracellular access is expected to obtain high-quality and long-term signal recording while maintaining cellular viability. The intracellular access should be achieved by the penetration methods that can be divided into spontaneous penetration and aided penetration. Spontaneous penetration mainly depends on cell phagocytosis and cell adhesion, while aided penetration requires external promoting factors such as chemical modification, electroporation or plasmonic optoporation.

### Spontaneous Penetration

**Fig. 3:**
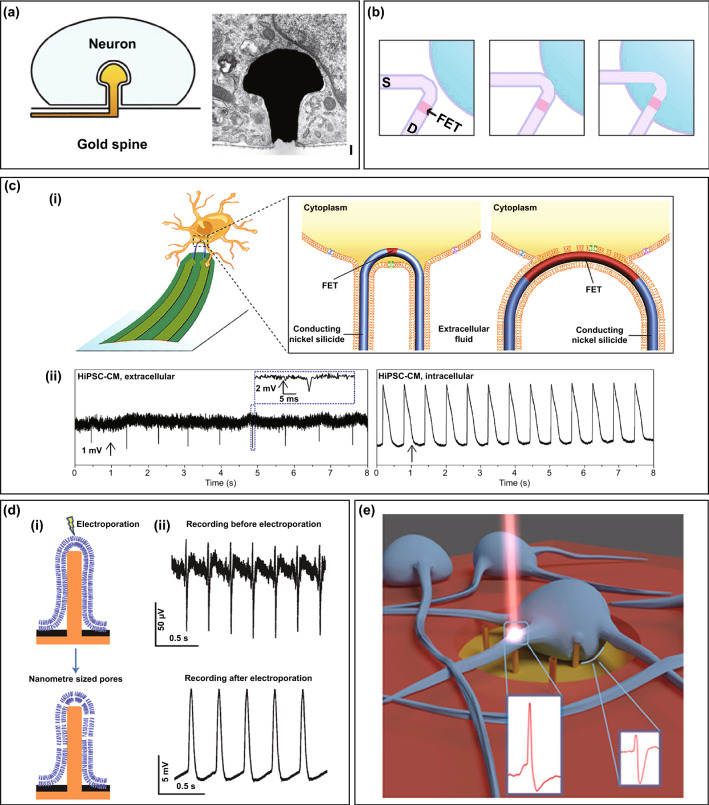
Methods for obtaining intracellular access. **a** Schematic illustration and SEM image of the neuron engulfing a gold-spine electrode. Reproduced with permission from Ref. [97]. Copyright 2010, Nature. **b** Schematic illustration of nanowire probe entrance into a cell. Dark purple and blue colours denote the phospholipid bilayers and cytosol, respectively. Reproduced with permission from Ref. [87]. Copyright 2010, American Association for the Advancement of Science. **c** (i) Schematic illustration of U-shaped nanowire probe with different radii of curvature (ROC) to permeate cellular membrane. (ii) Extracellular and intracellular recording from the nanoprobe with 1.5 μm ROC and 0.75 μm ROC, respectively. Reproduced with permission from Ref. [109]. Copyright 2019, Nature. **d** (i) Schematic illustration of the electroporation by a nanopillar electrode. (ii) The electrical signals recording before and after electroporation. Reproduced with permission from Ref. [91]. Copyright 2012, Nature. **e** Schematic illustration of plasmonic optoporation by MEA with 3D nanoelectrodes. Reproduced with permission from Ref. [100]. Copyright 2017, American Chemical Society

Spontaneous penetration takes advantage of natural cellular processes such as phagocytosis with less influence of native cell viability. Activation of their phagocytotic-like mechanisms is necessarily required, since cardiomyocytes and neurons present the strong capacity to internalize the foreign bodies as macrophages and endothelial cells, Hai et al. designed a functionalized gold-spine electrode (FGSE), which was modified by an engulfment-promoting peptide (EPP) with multiple Arg-Gly-Asp (RGD) repeats [97, 98], and the peptide effectively induced Aplysia californica neurons to actively engulf the gold-spine electrode to record the intracellular action potentials and subthreshold synaptic potentials (Fig. 3a). Moreover, induced phagocytosis can enhance the adhesion and electrical coupling of the cell–electrode interface, resulting in a high SNR that is comparable to conventional patch clamp recording.

Though the 3D spine electrode can achieve the intracellular access, the large dimension and geometry of electrodes potentially increase the difficulty of membrane penetration. To improve the efficiency of intracellular access, 3D nanowire electrodes were introduced due to their superiority of their sharp geometry and rigidity [9, 113, 135]. Two penetration mechanisms have been proposed, namely impaling as cells land onto the nanowire array and penetrating mediated by adhesive cells spreading on the substrate [136]. The former presents a low penetration probability by cell gravity, while the latter is more efficient under the action of adhesive forces to form a tight coupling between cells and the substrate. But in general, spontaneous penetration cannot satisfy efficient and stable intracellular recording.

### Aided Penetration

Spontaneous penetration possesses the superiority at intracellular access, however, only a fraction (< 7%) of 3D nanodevices can spontaneously penetrate cellular membranes [89, 137–139]. To enhance the penetration efficiency, aided penetration strategies are developed to achieve the intracellular access in recent decade, including chemical modification, mechanical forces, electroporation and plasmonic optoporation.

Chemical modification-aided penetration is based on the fusion of the coated phospholipid and the cellular membrane. Lieber group applied the phospholipid bilayer to a kinked SiNW, and a continuous shell was formed on the nanoprobes by fusion with unilamellar vesicles of phospholipid bilayers [1,2-dimyristoyl-sn-glycero-3-phosphocholine (DMPC)] (Fig. 3b) [87, 107, 109]. The lipid surface coating presents less effect on the conductance and sensitivity of nanodevices and possesses also less mechanical invasion to the cell, allowing for the recordings of robust intracellular potentials. By successful applications on kinked SiNW devices, the phospholipid modification was also applied for their nanotube field-effect transistors [102, 103]. It is worth noting that this penetration method possesses good reusability. When the nanoelectrode is retracted from the cell after the intracellular recordings, the nanostructure remains intact without blockage or damage; hence, it can gently recontact with the cell and repeat the high-quality intracellular recordings. To reveal the dimension effect of nanodevices on cell–device fusion, Zhao et al. designed a scalable U-shaped nanowire probe to explore the optimal structures and parameters of nanodevice for intracellular recordings [109]. Nanoscale membrane curvature will affect the endocytosis of cells. As the radii of curvature (ROC) of probe increased from 0.75 to 1.5 μm, the intracellular access based on endocytosis became weaker, and the amplitude of recorded action potentials decreased from ~ 34 to ~ 21 mV, respectively. Meanwhile, probe with 2 μm ROC can only perform the extracellular recording due to unsuccessful intracellular access (Fig. 3c). In addition, to trigger the spontaneous penetration, the biomimetic lipid bilayers of cell membrane were functionalized on the device surface as another effective chemical modification for aided penetration strategy [10, 140], which contains two hydrophilic ends and an intermediate hydrophobic band. Self-assembled monolayers were introduced to modify as the hydrophobic structure to achieve the fusion of cell membrane and functionalized electrode. Chemical modification penetrations are demonstrated to be minimally invasive, robust and reusable for intracellular recordings, but the chemical modification strategies still remain the low efficiency of intracellular access and modification processes are complicated. In addition, mechanically aided penetration is also another extensively intracellular access strategy, including pressing, micromanipulators or centrifugation [138, 139]. These studies highlight the potential of nanoelectrodes combined with physical forces to monitor intracellular activity.

To improve the efficiency of intracellular recording, the electroporation strategy is developed to achieve intracellular access. Because 3D nanoelectrode and the cell couple tightly and the surrounding electric field is localized at the tip of the electrode, the nanopores on the cell membrane generate at the cell–device interface with a low voltage (~ 1–3 V). A series of intracellular recording were based on nanoelectrode electroporation, and the signal amplitude is obviously enhanced while signal shape presents the intracellular feature with typical depolarization, repolarization and resting phases, demonstrating the access to intracellular environment (Fig. 3d). An alternating voltage pulse (0.5–1 V, 100 Hz, 300 ms) was applied to gold-mushroom-shaped microelectrodes (gMμEs) to achieve neuron electroporation, and the resting potential was recovered after a period of ~ 300 s [99]. Similarly, vertical SiNW and nanopillar devices have successfully accessed to the intracellular environment by administrating voltage pulses with amplitudes of ± 3 and ± 2.5 V on the cells, respectively [89, 91]. The intracellular potential recorded by nanopillar electrode arrays disappeared approximately 10 min after electroporation, and the duration was transient due to the resealing of cell membrane. To prolong the intracellular recording, Cui et al. developed a nanotube electroporation strategy, which achieved stable intracellular recording up to 1 h due to spontaneous infiltration of the cellular membrane into the nanotubes [101]. In addition, integrated current clamp is an effective strategy for electroporation and maintaining intracellular recording of neurons. Continued current injection can compensate for the current leakage from inside the cell in addition to maintaining membrane penetration, which is conducive to achieving sensitive subthreshold intracellular recording [94]. Though the electroporation strategy effectively improves the efficiency of intracellular recording (~ 100%), the tight cell-nanodevice coupling and seal are not essentially formed. Its instinct shortcomings and limitations should be urgently solved to ensure the native intracellular recordings. The electroporation pulse may interfere with the native cellular electrophysiological activities. Secondly, a recording interruption of ~ 10 s remains after electroporation to wait for recovery of amplifier saturation. Moreover, the duration and signal quality are still limited due to the transient nature of electroporation causes the recorded intracellular potential to be attenuated and thus provide no significant improvement on the electrode-cell interface.

To achieve the accurate and independent recording of cells in different regions, a plasmonic optoporation technique was developed [96, 100]. Dipalo et al. combined vertical nanodevices with plasmonic optoporation to achieve high signal-to-noise, long-term and stable intracellular recording (Fig. 3e) [100]. The nanoelectrode is stimulated by a short-pulse laser (8 ps, 1064 nm, 80 MHz) to open transient nanopores on the cell membrane. This process only occurs at the tip of the electrode, which has no effect on the cell–electrode seal and does not interfere with the spontaneous electrical activity. In contrast to electroporation, plasmonic optoporation does not require the passivation of planar electrodes, which can be used to simultaneously record intracellular and extracellular potential. Furthermore, the recording interruption is nonexistent in plasmonic optoporation, and consequently, continuous recording can be achieved. It is worth noting that the addressed plasmonic optoporation allows each nanoelectrode to be independently plasmonic and perforated independently, thus enabling the accurate selection of the cellular compartments for intracellular recording. However, plasmonic optoporation is difficult to perform high-throughput parallel regulation. 3D moving platform under the microscope is the only way to solve the multisite optoporation and realize the high-throughput precise regulation of cells. The development goal of aided penetration strategies is to combine the advantages of the approaches for achieving moderate, high-throughput, controllable and stable intracellular access.

## Principle and Simulation of Electrophysiological Recording

Action potentials or transmembrane potentials derive from the transfer of transmembrane ion currents of electrogenic cells [115, 141]. Intracellular ion concentration changes directly cause the fluctuation of transmembrane potential. Based on the defined recording and reference sites, the intracellular or extracellular potentials can be monitored. Two main types of nanodevices have been developed to detect electric signals: active field-effect transistor arrays that trigger gate signals through surface potential changes and passive electrode arrays that induce currents through interface impedance. By advanced micro/nanofabrication technologies, the versatile 3D nanoscale multitransistor and multielectrode are manufactured as the powerful tools to collect the electrophysiological information of cells from the extracellular to the intracellular.

### Active Field-effect Transistor Recording

**Fig. 4:**
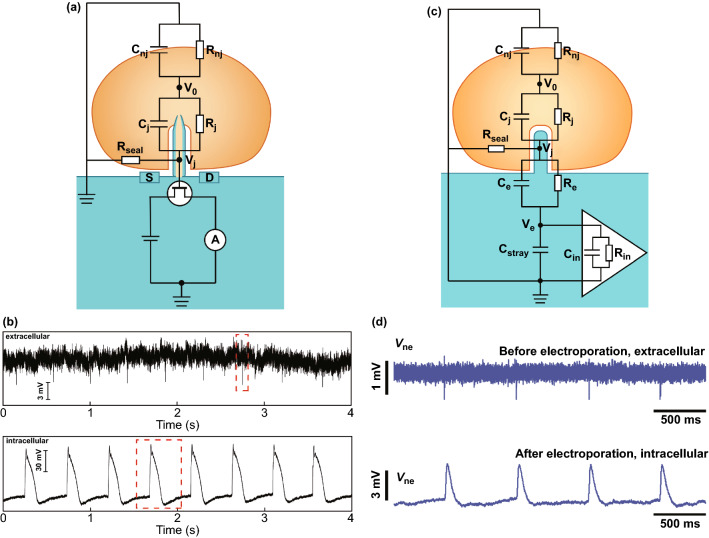
The equivalent circuit for simulating cell-nanodevice coupling. **a** Circuit model for describing the active field-effect transistor recording. **b** Extracellular and intracellular recording by active field-effect transistor. Reproduced with permission from Ref. [87]. Copyright 2010, American Association for the Advancement of Science. **c** Circuit model for describing the passive nanoelectrode array recording. **d** Extracellular and intracellular recording by passive nanoelectrode array. Reproduced with permission from Ref. [92]. Copyright 2017, Nature

The field-effect transistor is conventionally fabricated on a semiconductor substrate, a source, a drain and a gate electrode. In a conventional FET, the gate metal electrode is used to apply a gate voltage (V_g_) [142]. When Vg exceeds the threshold voltage, electrons or holes are induced to move towards the semiconductor oxide interface, lowering the channel barrier and generating a significant tunnelling current. For the electrophysiological recording, the effective potential generated by action potential is applied on the gate electrode, which can regulate the conductance and tunnelling current between the source and drain electrodes. However, the source/drain (S/D) electric contacts for current injection and collection greatly limit the device to achieve the intracellular access. To solve this problem, Lieber et al. designed a 3D kinked nanowire probe, making a significant breakthrough for intracellular recording [87]. A FET sensitive region is defined at the tip of the kinked nanowire entering the intracellular, while the S/D structure remains extracellular. On this basis, the branched intracellular nanotube FET (BIT-FET) has been subsequently developed, where a branched hollow SiO_2_ nanotube penetrates the membrane as the 3D bioprobe of FET gate, and the S/D is positioned on the extracellular Si nanowire [102]. In particular, for p-type FETs, the negative gate voltage causes carrier accumulation and conductivity to increase, while the positive gate voltage causes carrier depletion and conductance to decrease; hence, the recorded conductance is inversely proportional to potential on the gate electrode, and intracellular potential recorded by nano-FET device is independent of the interface impedance, which effectively avoids the signal loss caused by the electrode impedance and can achieve high-fidelity intracellular recording [107, 109, 143]. To visually describe the FET recordings, an equivalent circuit is applied to simulate the cell-FET coupling (Fig. 4a). V0 refers to action potential of cell, *C*_nj_ and *R*_nj_ refer to nonjunctional capacitance and resistance, respectively, *C*_j_ refers to junctional capacitance, which consists of cell capacitance, nanotube capacitance and bilayer capacitance, *R*_j_ refers to junctional resistance, and Rseal refers to seal resistance. When an action potential occurs, *V*0 diffuses from the intracellular region. The gate potential is equal to the junctional potential *V*_j_, which can be calculated as follows:

1 $${V}_{j}={V}_{0}\cdot \frac{{R}_{\mathrm{seal}}}{{Z}_{j}+{R}_{\mathrm{seal}}}$$

2 $${Z}_{j}=\frac{{R}_{j}}{1+i\omega {C}_{j}{R}_{j}}.$$

*Z*_j_ refers to junctional impendence, and ω refers to angular frequency of potential. The variation in *V*_j_ can regulate the conductance of the FET from source to drain electrodes. The cell–electrode coupling of an FET can be evaluated by the ratio between *V*_j_ and *V*_0_; a higher ratio refers to better coupling and thus better recording capability.

According to Eq. (1), the coupling is dependent on *Z*_j_ and Rseal. *Z*_j_ presents to the interface impedance from the action potential to the FET gate. Rseal presents to the seal capability after cell adhesion, and a tighter adhesion on the device leads to a better seal. Cell–electrode coupling can be observed by recording the results. Figure 4b shows the extracellular and intracellular recording from the same cardiomyocyte by FET probe [87]. In the extracellular recording, the cell membrane had not been punctured, so *Z*_j_ expressed high impedance. In the intracellular recording, *Z*_j_ was close to the zero due to the direct contact between the cytoplasm and FET gate after penetration.

### Passive Electrode Arrays Recording

Since Thomas et al. pioneered the use of microelectrode array technology to detect extracellular signals of isolated chicken embryonic cardiomyocytes, various types of MEAs have been developed to record the electrophysiological activities of cells [29, 32, 88, 144–146]. In MEAs, metal microelectrodes, leads and passivation layers are fabricated on an insulating substrate by various fabricating processes. The electrode is a bridge between the cell and the peripheral circuit, one end is in contact with the cell to receive a weak electrical signal, while the other is connected to an amplifier. After the cell couple on the electrode, the cell–electrode interface reaches electrochemical equilibrium to form a double layer. The electrode surface produces polarization, and the double-layer capacitor charges and discharges to detect the fluctuation of surface potential induced by action potential of cell. The electrode is usually connected to an external amplifier by metal traces covered with dielectric materials for signal processing, which is suitable for the study of single-cell potential changes and multicellular network transmission both in vivo and in vitro [20, 41, 147–149]. To improve the cell–electrode coupling, 3D nanoelectrode arrays were developed for intracellular recordings with the advantages of minimal invasion and high spatial resolution, the layout complexity of interconnects hampered the density and resolution of nanodevices, besides the increase in electrode impedance significantly raises noise and lower the signal amplitude [93, 150]. To overcome this constraint, CMOS technologies are introduced for nanodevice fabrication. The electronic components for amplification and stimulation were integrated on a CMOS circuit, so the nanodevices directly located above the circuit to reduce the layout difficulty and interconnect impedance between the electrodes and exterior condition circuits [92, 94]. The active nanoelectrode-based CMOS device combines the advantages of passive electrode and active electrode, and has a prominent superiority in achieving large-scale cell network recording.

To visually describe the passive nanoelectrode array recording, an equivalent circuit of passive nanoelectrode array is shown in Fig. 4c. The equivalent model of cell–electrode is the similar as the FET case, while the junctional voltage *V*_j_ is different that serves as the gate voltage in the FET. In the nanoelectrode array equivalent circuit, *V*_j_ conducts further to Ve and is then recorded by the amplifying circuit. The junctional potential *V*_j_ can be calculated as follows:

3 $${V}_{j}={V}_{0}\cdot \frac{{Z}_{\mathrm{sys}}}{{Z}_{j}+{Z}_{\mathrm{sys}}}$$

4 $${Z}_{j}=\frac{{R}_{j}}{1+i\omega {C}_{j}{R}_{j}}$$

5 $${Z}_{\mathrm{sys}}={R}_{\mathrm{seal}}\cdot \frac{{Z}_{\mathrm{amp}}+{Z}_{\mathrm{e}}}{{R}_{\mathrm{seal}}+{Z}_{\mathrm{amp}}+{Z}_{\mathrm{e}}}$$

6 $$ Z_{{\text{e}}} = \frac{{R_{{\text{e}}} }}{{1 + i\omega C_{{\text{e}}} R_{{\text{e}}} }} $$

7 $$ Z_{amp} = \frac{{R_{in} }}{{1 + i\omega R_{in} \left( {C_{stray} + C_{in} } \right)}} $$

where *Z*_sys_ the system impedance, consisting of a hybrid junction with a seal resistance (*R*_seal_), electrode impedance (*Z*_e_) and amplifier impedance (*Z*_amp_). *R*_e_ and *C*_e_ refer to electrode resistance and capacitance, respectively. *R*_in_ and *C*_in_ refer to the amplifier input resistance and capacitance, respectively. Cstray is the stray capacitance.

Consequently, the electrode voltage (*V*_e_) can be written as:

8 $$ \begin{aligned} V_{{\text{e}}} & = V_{{\text{j}}} \cdot \frac{{Z_{{{\text{amp}}}} }}{{Z_{{\text{e}}} + Z_{{{\text{amp}}}} }} = V_{0} \cdot \frac{{Z_{{{\text{sys}}}} }}{{Z_{{\text{j}}} + Z_{{{\text{sys}}}} }} \cdot \frac{{Z_{{{\text{amp}}}} }}{{Z_{{\text{e}}} + Z_{{{\text{amp}}}} }} \\ & = V_{0} \cdot \frac{{R_{seal} \cdot Z_{amp} }}{{Z_{{\text{j}}} \left( {R_{{{\text{seal}}}} + Z_{{{\text{amp}}}} + Z_{{\text{e}}} } \right) + R_{{{\text{seal}}}} \cdot \left( {Z_{{{\text{amp}}}} + Z_{{\text{e}}} } \right)}}. \\ \end{aligned} $$

The cell–electrode coupling efficiency of an MEA can be defined as the ratio of *V*_e_ to *V*_0_. From the derivation of Eq. (8), higher seal resistance and amplifier impedance are beneficial to improve the signal quality, while junction impedance and electrode impedance are contrary.

System impedance *Z*_sys_ is related to seal resistance, amplifier impedance *Z*_amp_ and electrode impedance *Z*_e_; as shown in Eq. (5), electrode impedance is expected to be low. Amplifier impedance mainly depends on the input resistance *R*_in_ of the amplifier. Theoretically, larger input resistance results in larger amplification and higher coupling efficiency. However, limited by the input power of the amplifier, the input resistance has an upper limit, which depends on the amplifier type. Seal resistance *R*_seal_ depends on the adhesion between the cell and the electrode. Junction impedance *Z*_j_ is close to zero by achieving the puncture of the cell membrane (Fig. 4d). Therefore, the supplementation of electroporation or plasmonic optoporation for intracellular recording can obviously enhance the coupling and intracellular signals [91, 100].

For instance, the 3D mushroom electrode obviously increased the seal resistance from 1 to 100 MΩ compared to the planar electrode [97, 151]. The application of a 3D nanowire array and hollow nanotube array further improved the seal resistance [89, 101]. These were ascribed to the bionic interface of the vertical structure, which provided an excellent microenvironment for cell adherence. On the other hand, a single vertical structure led to high electrode impedance because of the decrease in the cross-sectional area [97]. Overall, the application of 3D nanodevice can achieve a remarkable cell–electrode coupling efficiency for intracellular recordings. To improve the performance of passive nanodevices mainly includes two aspects: (1) Enhancing the seal resistance between the cell and the electrode to minimize signal loss to the bath medium. Optimizing the three-dimensional geometries of nanodevices can improve the bio-interface coupling. (2) Reducing the cell-interface impedance to increase signal collection efficiency. Effective aided penetration strategy is beneficial for decrease in cell-interface impedance. Through the continuous optimization of passive devices, it is expected to replace active ones, becomes a powerful tool for high-fidelity and high-throughput electrophysiology research.

## In-Cell Nanoelectronics for Cardiology and Neuroscience

A variety of nanodevices have been developed to obtain intracellular potentials (Table 1). Based on their characteristics, applying nanodevices to basic active units (cardiomyocytes and neurons) can enable breakthroughs in the field of cardiology and neuroscience. Intracellular recording can get closer to the true action potential to explore the mechanisms of ion channels with the high-resolution detail for modelling diseases and screening drugs. Second, intracellular recording possesses high sensitivity, which is also conducive to the study of subthreshold potential or membrane oscillations and is of great significance to the exploration of the activities of cell network. Finally, intracellular recording enables one-to-one correspondence between cells and electrodes, which lays a foundation for high-precision recording and the manipulation of individual target cells. The amplitude, shape and duration are key parameters to assess the performance of intracellular recording, so optimizing the cell–electrode interface and effective aided penetration strategy are potential strategies to promote one or all of these parameters for long-term chronic practical applications.

**Table 1:** Summary of in-cell nanoelectronics for cardiology and neuroscience

Cell type	Material	Geometry	Process	Complexity	Throughput	Cost	Access strategies	Amplitude	SNR	Duration	Refs
Embryonic chicken cardiomyocytes	Si	Kinked nanowire	VLS, EBL	High	1	High	Phospholipid bilayers coating	~ 80 mV	N/A	N/A	[87]
Embryonic chicken cardiomyocytes	Si, SiO_2_	Nanowire with branched nanotube	VLS, EBL, ALD, H_2_O_2_ etching	High	1	High	Phospholipid bilayers coating	75–100 mV	N/A	N/A	[102]
Embryonic chicken cardiomyocytes	Si	Kinked nanowire	VLS, CVD, EBL	High	1	High	DMPC bilayer modifying	60 mV	N/A	N/A	[104]
Rat ventricular myocytes	SiO_2_ coated with Pt	Vertical nanowire	PECVD, metal sputtering, photolithography, sputtering, ALD	High	1024	High	Electroporation	5–20 mV	N/A	5–20 min	[92]
HL-1 cardiomyocytes	Pt	Nanopillar	FIB, PECVD, photolithography	Low	60	Low	Electroporation	11.8 mV	590	~ 10 min	[91]
HL-1 cardiomyocytes	IrOx	Nanotube	Electrodeposition, EBL, PECVD, photolithography	Low	60	Low	Electroporation	1.5–15 mV	N/A	~ 60 min	[101]
Human cardiomyocytes	Pt	Nanopillar	FIB, PECVD, EBL and photolithography	Low	60	Low	Electroporation	25.15 mV	838	N/A	[95]
Primary rat cardiomyocytes	Pt–Ti–Au-Ti-SiO_2_	Nanovolcano	E-beam evaporation and EBL	Low	32	Low	Monolayers of alkanethiols modifying	~ 20 mV	N/A	~ 70 min	[10]
Human cardiomyocytes, HL-1 cardiomyocytes	Au, Si_3_N_4_	Nanotube	E-beam evaporation, FIB lithography	Middle	24	Middle	Electroporation	~ 1.5 mV	N/A	10–15	[152]
Hippocampal neurons, HL-1 cardiomyocytes	Au	Nanocylinder	FIB, thermal evaporation	Low	N/A	Low	Plasmonic optoporation	~ 1.5 mV	N/A	~ 80 min	[100]
Rat dorsal root ganglion neurons	Si	U-shaped nanowire	VLS, EBL, ALD, photolithography	High	1–2	High	Phospholipid bilayers coating	60–100 mV	115 ± 29	N/A	[109]
Snail buccal neurons	Au	Mushroom-shaped spine	Electroplating, photolithography, CVD	Low	62	Low	Engulfment-promoting peptide coating	0.1–25 mV	N/A	N/A	[97]
Rat cortical neurons	Si coated with Au	Vertical nanowire	EBL, RIE, ALD, thermal or e-beam evaporation, photolithography	Middle	16	Middle	Electroporation	~ 4 mV	> 100	N/A	[89]
Rat neurons	Si coated with Pt	Vertical nanoneedle	CVD, EBL, ALD, electrodeposition, photolithography	High	4096	High	Electroporation	~ 4 mV	N/A	Median ~ 8 min	[94]
Mouse, rat and human neurons	Si	Vertical nanowire	EBL, RIE, PECVD, photolithography, SF6 inductively coupled plasma	High	64	High	Spontaneous penetration	~ 99 mV	1700	N/A	[9]

### In-Cell Nanoelectronics for Cardiomyocytes

Cardiomyocytes are the main bioactive and functional components of the heart, and their unique membrane structure is composed of a phospholipid bilayer and membrane proteins, which can not only control ion transport to maintain the homeostasis of intracellular environment but also provide varieties of specific ion channels as the basis to generate action potentials [153, 154]. According to the characteristics of potential, the transmembrane potential of cardiomyocytes can be divided into resting potential and action potential. When cardiomyocytes are in the “resting” state, the cellular membrane is polarized, with transmembrane potential of ~ -90 mV [155, 156]. The action potential is spontaneously generated by autonomic cardiomyocytes (e.g. sinoatrial node cells), and then is transmitted to working cardiomyocytes (e.g. atrial myocytes and ventricular myocytes) [157, 158]. The action potential of working cardiomyocytes can be mainly divided into five phases: rapid depolarization (Na^+^ inflow), early rapid repolarization (K^+^ outflow), plateau (K^+^ outflow, Ca^2+^ inflow), late rapid repolarization (K^+^ outflow) and resting state (Na^+^, K^+^, Ca^2+^ concentration recovered by ion pump) [159–161]. During the whole process, the action potential measured by patch clamp usually possesses an amplitude of ~ 100 mV and a duration of ~ 200–400 ms [156, 160]. It is worth noting that the shape of action potential will be affected by cell types, such as pacemaker, ventricular myocytes and atrial myocytes, which are different in the duration, the refractory period, etc. [95, 156]. In contrast to working cardiomyocytes, sinoatrial node cells have a depolarization (Ca^2+^ inflow), then turn into repolarization (K^+^ outflow) without a plateau. In addition, the high seal resistance and low interface impedance can improve the signal quality [162].

**Fig. 5:**
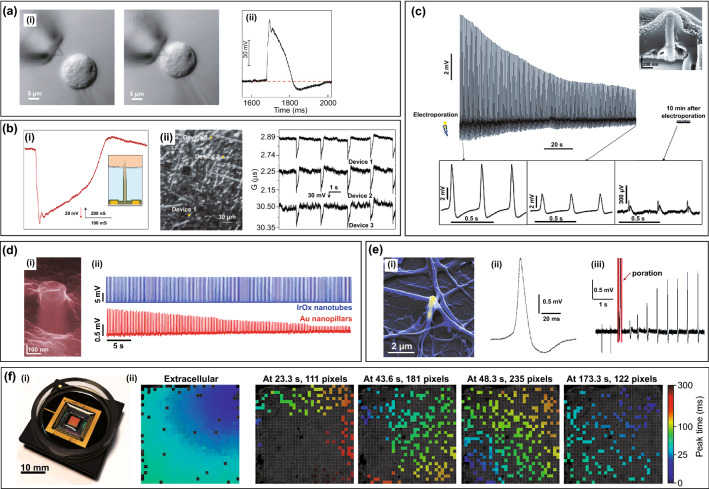
In-cell nanoelectronics for cardiomyocytes. **a** (i) HL-1 cell approach, contact and internalized kinked nanowire probe. (ii) Intracellular recording by kinked nanowire FET. Reproduced with permission from Ref. [87]. Copyright 2010, American Association for the Advancement of Science. **b** (i) Intracellular recording by branched nanotube FET. (ii) Synchronous intracellular recording by three BIT-FET devices. Reproduced with permission from Ref. [102]. Copyright 2011, Nature. **c** Intracellular recording by nanopillar electrodes. The amplitude decays within 10 min after electroporation. The inserted image (upper right) shows the nanopillar electrode being fully engulfed by the cell. Reproduced with permission from Ref. [91]. Copyright 2012, Nature. **d** (i) IrOx nanotube device engulfed by cardiomyocytes. (ii) Intracellular recording by IrOx nanotube electrodes. The action potential remained stable within 1 min compared with Au nanopillar electrodes. Reproduced with permission from Ref. [101]. Copyright 2014, Nature. **e** (i) Gold plasmonic 3D nanoelectrodes enveloped by cell. (ii) Intracellular recording by nanoelectrodes after plasmonic optoporation. (iii) The gradually increased amplitude of action potentials within the first 2 s after plasmonic optoporation. Reproduced with permission from Ref. [100]. Copyright 2017, American Chemical Society. **f** (i) The device of nanodevices combining with CMOS circuit. (ii) The spatial propagation of action potentials before and after electroporation. Reproduced with permission from Ref. [92]. Copyright 2017, Nature

To date, intracellular recording based on 3D nano-FET devices comes the closest to the true action potential. Kinked nanowire FETs were gently exposed to spontaneously beating embryonic chicken cardiomyocytes, and after an interval of ~ 40 s, the initial 3–5 mV extracellular signal was gradually transformed into “action potential” signal with larger amplitude and opposite polarity (Fig. 5a) [87]. This intracellular signal possessed an amplitude of ~ 80 mV and a duration of ~ 200 ms, and the characteristic phases of the action potential can be obviously distinguished. Similarly, single branched intracellular nanotube FET (BIT-FET) also recorded high-quality action potential with 75–100 mV amplitude and ~ 200 ms duration from cardiomyocytes (Fig. 5b, i) [102]. However, the complexity of FET significantly hampered the large-scale manufacturing, and their noise is too large to accurately record the weak potential and resting potential without stimulation function. To overcome these shortcomings, increasing attention has been paid to intracellular recordings based on vertical electrodes. Cui et al. recorded an action potential of 11.8 mV from electroporated HL-1 cells by nanopillar electrodes (Fig. 5c) [91]. Compared with nano-FET devices, the amplitude decreases, but the SNR increases. The details of the high-resolution HL-1 action potential can be used to distinguish the (non-) pacemaker cells and verify the shortening and prolongation of the action potential by typical ion blockers of nifedipine and tetraethylammonium, respectively. For human embryonic stem cell-derived cardiomyocytes (hESC-CMs), nanopillar electrodes can measure an intracellular action potential of 25.15 mV [95]. The shape of action potential can be used to distinguish all three cardiomyocyte subtypes, and the parameters of signals are useful for ion channel drug screening and heart disease modelling. However, the potential recorded by nanopillar electrodes gradually decays within 10 min and rapidly turns to extracellular potential, which limits its application in long-term recording (Fig. 5c). The IrOx nanotube electrode was optimized against this constraint, which could record the intracellular potential of ~ 15 mV and maintain long-term (~ 1 h) intracellular access after electroporation (Fig. 5d) [101]. By combining the tubular structure with plasmonic optoporation, tighter intracellular coupling can be achieved, and the peak amplitude can remain stable for 20–30 min, even more than 80 min (Fig. 5e) [100]. In particular, it can be observed that the amplitude gradually increases after plasmonic optoporation, which is beneficial to study the formation mechanism of the cell–electrode interface. Furthermore, the electrode structure of a nanovolcano combined with chemical modification can achieve intracellular potential with an amplitude of 20 mV while allowing for up to an-hour stable recordings [10].

The scalable and minimally invasive characters of nanodevices play crucial significant role for the synchronous long-term monitoring of cardiomyocytes. The scalability of nanodevices was tested on nano-FET devices [102, 103]. Two or three independent BIT-FETs were used to simultaneously measure cells at the same or different locations to obtain full-amplitude intracellular potentials, which demonstrated their potential for network-level multiplexed measurements (Fig. 5b, ii) [102]. Due to technological diversity and structural flexibility, more attempts have been made with vertical nanodevices to achieve multiplexed and long-term monitoring. Cui et al. conducted two experiments, including simultaneous intracellular recording with five different nanopillar electrodes and monitoring for three consecutive days before and after electroporation, and the action potentials with similar shape and duration were obtained [91]. Similarly, simultaneous recording with six different IrO_x_ nanotube electrodes revealed that synchronous activities of the cultured cells and continuous monitoring for 8 days could directly reflect the maturation and ageing of cells [101]. The scalability of nanodevices has been greatly improved by combination with CMOS circuits. A total of 1,024 “pixels” equipped with vertical nanowire electrodes were used to simultaneously detect hundreds of connected cardiomyocytes [92]. The results showed that 968 pixels were coupled with cells, and the spatial propagation of action potential could be clearly observed before and after electroporation (Fig. 5f). The CMOS nanoelectrode array possessed obvious advantages in studying the network propagation of membrane potential and detecting drug effects at the single-cell and cell network levels, respectively.

### In-Cell Nanoelectronics for Neurons

Neurons are basic units of nervous organs or tissues, such as brain, spinal cord, retina. Excitability and conductivity are also the main functions of neurons. The electrical signals produced by neurons are similar in principle and waveform to those of cardiomyocytes [80, 163]. The resting potential of neurons is generally in the range of -30 to -90 mV; when stimulated, the negative potential raises rapidly and reached + 30 mV. The action potentials of neurons have the following characteristics: (1) The waveform and amplitude of action potentials produced by the same cell remain the same during signal transduction even if the conduction distance and stimulus intensity change [164–166]. (2) The transduction of action potentials on the same neuron is full-amplitude, active and long-distance without signal attenuation. (3) The signal superposition of action potential signal is nonexistent because neurons possess a refractory period so the excitability of can vary with the transmembrane potential. These characteristics lay a foundation for studying neural connections and achieving network mapping based on electrophysiological recording. In contrast to the cardiomyocyte, stable intracellular recordings of neurons are more difficult due to the cell connection. The cultured cardiomyocytes can fuse and connected with each other through electrical gap junctions, while the neurons are mainly connected through the chemical synapses with few electrical connections, so they are electrically isolated. Therefore, the neuronal intracellular recording is much more challenging than the cardiac intracellular recording. Many nanodevice research has been very successful in intracellular recording of cardiomyocytes, but most of them have the difficulty in intracellular recording of neurons. Though these nanodevices coupled intracellularly with neurons, most of them still failed to record subthreshold PSPs (± 0.5–10 mV) with few successful works [94], which applied the advanced integrated current clamp electronics.

**Fig. 6:**
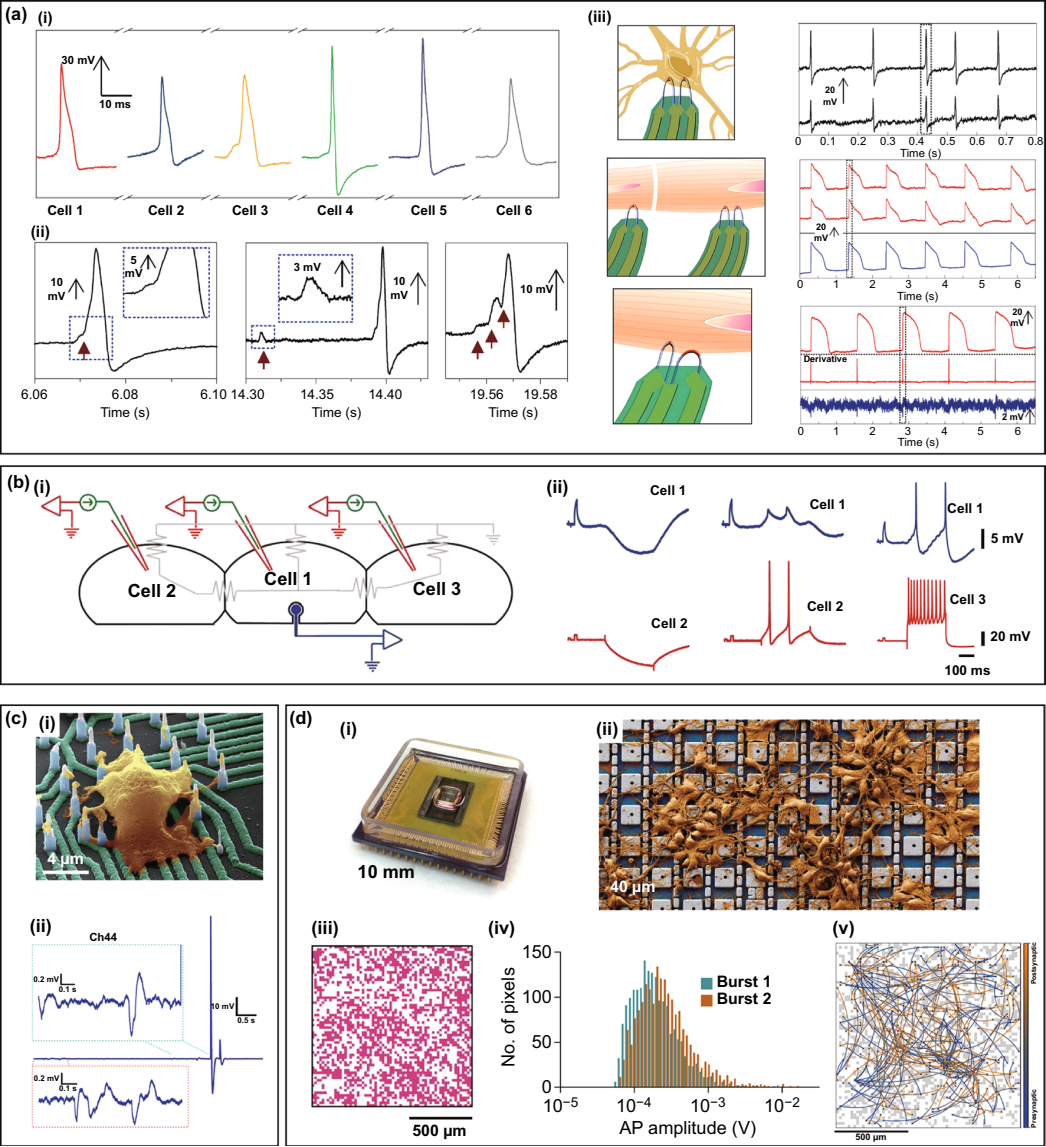
In-cell nanoelectronics for neurons. **a** (i) The sequential recording of six independent dorsal root ganglion (DRG) neurons by U-NWFET. (ii) Three different subthreshold activities of DRG neurons measured by U-NWFET. (iii) The multiplexing of U-NWFET. Multiple U-NWFET probes were used to simultaneously measure a single neuron (top), different neurons (middle) and intracellular/extracellular potentials (bottom). Reproduced with permission from Ref. [109]. Copyright 2019, Nature. **b** (i) Three buccal neurons cultured on FGSEs. (ii) Synaptic potentials recorded from neuron 1 by a FGSE after the depolarizing or hyperpolarizing pulses were applied to neuron 2 or 3. Reproduced with permission from Ref. [97]. Copyright 2010, Nature. **c** (i) hiPSC-derived cortical neurons cultured on nanowires. (ii) Intracellular recording of a 99 mV action potential and subthreshold oscillations based on the individually addressable nanowire electrode. Reproduced with permission from Ref. [9]. Copyright 2017, American Association for the Advancement of Science. **d** (i) The device of CMOS-activated 4096 nanoelectrode array. (ii) Neurons cultured on top of the electrode array. (iii, iv) Network-wide intracellular recording of rat cortical neurons with about 44% cells burst (iii) and the median amplitude of ~ 200 μV (iv). (v) 304 synaptic connections mapped by a nanoelectrode array. Reproduced with permission from Ref. [94]. Copyright 2020, Nature

The amplitude and SNR of electrical signals are key characters to evaluate the quality of intracellular recordings, and the true and clear recording of action potentials (APs) and postsynaptic potentials (PSPs) is of great significance for neural network investigation. Nano-FET devices possess unique advantages in intracellular recording. A U-shaped nanowire FET (U-NWFET) designed by Zhao et al. was used to synchronously measure six individual dorsal root ganglion (DRG) neurons, and potentials with consistent shape and duration were observed with an amplitude of 60–100 mV (Fig. 6a, i) [109]. In contrast, electrical signals measured by vertical nanowire electrodes suffer from partial signal loss, and the amplitudes reached up to 20 mV [89, 94, 97, 100, 113]. However, Liu et al. designed an individually addressable nanowire electrode, which for the first time measured action potentials of 99 mV, comparable to those measured by a patch clamp (Fig. 6c) [9]. For the SNR, the intracellular recording by U-NWFET can reach 115 ± 29, which allows observation of 3 ~ 5 mV subthreshold signals (Fig. 5a, II), demonstrating its potential for measuring postsynaptic potentials [109]. In addition, functionalized gold-spine electrodes (FGSEs) were used to record excitatory postsynaptic potentials from three buccal neurons (Fig. 6b, i) [97]. The depolarizing or hyperpolarizing pulses were applied to adjacent cells, and consistent potentials were eventually recorded on the intermediate cells by FGSE (Fig. 6b, ii). Other vertical nanowire/nanotube electrodes achieve the high SNR neuronal intracellular recordings [9, 94, 100], and only CMOS-activated 4096 nanoelectrode arrays record PSPs and define characteristic of the PSPs [94]. Robinson et al. averaged the waveforms obtained under the same experimental conditions, improving the SNR from ~ 100 to > 1000 [89]. In particular, the individually addressable nanowire electrode achieved a SNR of 1700 based on a large aspect ratio, which provided a larger interaction region for neurons. The detailed presentation of action potentials with a high SNR allows for signalling blocker screening and dopaminergic neuron modelling [9].

The multiplexing of nanodevices is another technique used to realize the mapping of neural networks. Multiple U-NWFET probes were used to simultaneously measure the same/different DRG neurons (Fig. 6a, iii) [97]. The results showed that there was no obvious delay or waveform difference between the two channels from the same cell, and the delay of signals between different cells was consistent with the propagation speed of neuron potentials. In particular, two U-NWFET probes with different curvatures were integrated on the same substrate, providing a new concept for the simultaneous measurement of intracellular and extracellular potentials. Multiple vertical nanotube/nanowire electrodes were also implemented to simultaneously record the intracellular potentials of neurons, thus demonstrating their multiplexing potential [9, 89, 94, 100]. Abbott et al. integrated 4096 nanoelectrodes (Fig. 6d, i, ii), which could be coupled with approximately 44% of cultured rat cortical neurons, and could record action potentials with a median amplitude of ~ 200 μV (individual amplitudes could reach ~ 10 mV) (Fig. 6d, iii, iv). In addition, the electrode array can record the transmission of synaptic potential and the burst of action potentials by further quantifying the amplitude of synaptic potential, and 304 synaptic connections can be mapped from the 19-min long intracellular recording data of 1728 neurons (Fig. 6d, v) [94].

### Multifunctional In-cell Nanoelectronics

With the development of nano/microfabrication and bioelectronics, more attention has been paid to the multifunctional nanodevices. In addition to single intracellular recording, nanodevice can be applied to cell stimulation, biological/chemical sensing and intracellular delivery. The multifunctional nanodevices not only improve the practice but also expand the application prospects in molecular detection, drug screening and disease modelling.

**Fig. 7:**
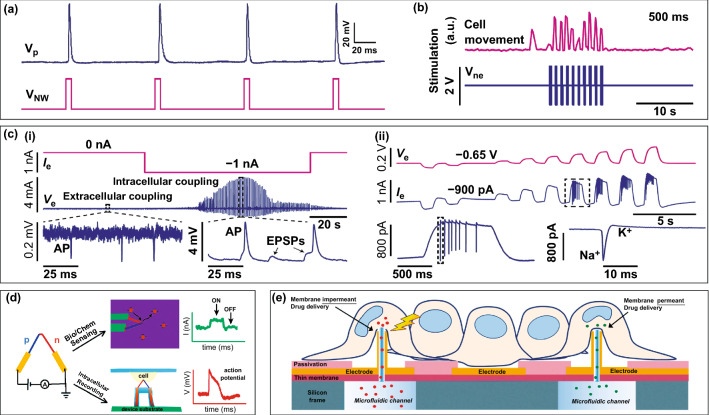
Multifunctional in-cell nanoelectronics. **a** Voltage pulses injected through vertical nanowire electrodes to induce action potentials of neurons. Reproduced with permission from Ref. [89]. Copyright 2012, Nature. **b** Current pulses injected through vertical nanowire electrodes to change the beating frequency of cardiomyocytes. Reproduced with permission from Ref. [92]. Copyright 2017, Nature. **c** (i) Current injection and potential recording in the pCC configuration. (ii) Voltage injection and current recording in the pVC configuration. Reproduced with permission from Ref. [94]. Copyright 2020, Nature. **d** Schematic illustration of p–n diode device for intracellular recording and bio/chem sensing. Reproduced with permission from Ref. [104]. Copyright 2012, American Chemical Society. **e** Schematic illustration of the biosensor for selective intracellular delivery and intracellular recordings. Reproduced with permission from Ref. [152]. Copyright 2018, Royal Society of Chemistry

To decipher the functional mechanisms of the heart and brain, simultaneous intracellular recording and stimulation are necessary. As a bidirectional connection between cells and circuits, nanodevices can not only receive signals from the cell but also output electrical stimulation to the cell. Park's team designed a series of vertical nanowire electrodes and demonstrated that injecting current/voltage pulses into neurons/cardiomyocytes through them can induce action potentials or change the firing frequency (Fig. 7a, b) [89, 92]. By applying a bias (~ -1.5 V) to the nanowires, changes in the cellular membrane potential can be recorded, but the change only existed locally due to the microscale point source stimulation provided by the nanowires. In particular, an electrical signal recording device integrated with 4096 nanoelectrodes can be configured in either pseudocurrent-clamp (pCC) mode or pseudovoltage-clamp (pVC) mode [94]. In the pCC configuration, the circuit has a high input impedance that allows electroporation and potential recording to be concurrent, and continued current injection can compensate for the current leakage from inside the cell in addition to maintaining membrane penetration (Fig. 7c, i). In the pVC configuration, the circuit is converted to low input impedance, which allows simultaneous voltage injection and current recording and is suitable for neuron ion channel activation and current measurement (Fig. 7c, ii).

The nanoscale p–n diode device was developed with the dual functions of intracellular recording and biochemical sensing (Fig. 7d) [104]. First, the device employed a nanostructure and membrane penetration strategy which is similar to the kinked nanowire FET and can record an intracellular action potential of over 60 mV. Second, p–n nanowire probes can be coupled to poly(dimethylsiloxane) (PDMS) microfluidic channels to detect fluorescently charged polystyrene nanobeads in aqueous solution. When the negatively charged nanobead approaches and/or adheres to the p–n junction, the conductance of the device increases, and the motion of the nanobeads can be further correlated with the conductance changes by combining with confocal imaging. Moreover, optimization and adjustment of the relative doping ratio in the p/n region can expand the potential of p–n nanowire probes for highly localized sensing in different fields. In addition, 3D plasmonic nanostructures combined with multielectrode arrays can achieve electrophysiological measures of large networks and simultaneous chemical analyses by Raman spectroscopy [167]. Another design of the multifunctional nanoelectrode is to combine hollow vertical nanostructures with microfluidic channels at the bottom (Fig. 7e). These nanoelectrodes can selectively deliver a variety of molecules to targeted cells and simultaneously monitor the electrical activity of cardiomyocytes/neurons, which provides new methodologies for early pathological studies [152, 168].

## Summary and Perspective

The advances of in-cell nanoelectronics are of great significance to achieve the intracellular recordings (Table 1). First, regarding the design of 3D nanodevices, the combination of semiconductive and metal materials improves the performance of intracellular recordings and is compatible with CMOS technology; the nanoscale dimension of devices enables intracellular recording in a minimally invasive manner; the vertical nanotube/nanovolcano structure increases the curvature of cell membrane and prolongs the duration of intracellular recordings; and advances in top-down and bottom-up processing approaches lay the foundation for the development and large-scale manufacturing of in-cell nanodevices. Second, in terms of intracellular access, various aided penetration strategies have significantly improved the penetration efficiency of nanodevice and allowed us to derive the stable intracellular action potentials. Finally, in terms of electrode-cell coupling, nanodevices based on active multitransistor devices are independent of the interface impedance and can record the intracellular potential with fully amplitude; while the passive multielectrode devices possess a high SNR and can be integrated with high spatial resolution, addressable, high-throughput CMOS circuits. Based on the superiority of in-cell nanoelectronics, nanodevices are minimally invasive, accurate, sensitive and stable; they can multiplex the intracellular recordings of cardiomyocytes and neurons and have been widely used in drug screening, disease modelling and cell network mapping. Multifunctional nanodevices have also been developed to allow simultaneous electrophysiological recording, cellular stimulation, biological/chemical sensing or drug delivery, providing a powerful platform for mechanism research and disease treatment in the fields of cardiology and neuroscience.

Looking forward, many challenges and potentials remain in developing and improving nanoelectronics for intracellular recording, particularly in neuroscience. First, it is necessary to optimize the structure, improve the performance and simplify the manufacturing process of the nanodevice. Sensitivity is important to disclose a great deal of information contained in subthreshold activities (e.g. PSPs) for exploring the neuron circuit behaviour and the change in the action potential configuration; scalability makes sense for improving efficiency and reducing cost in commercial and clinical applications. Currently, only a few CMOS MEA devices have enabled to synchronously achieve high-fidelity and large-scale intracellular recording [92, 94, 96]. Electrophysiological detection based on mirror charge that transducing cell ionic currents into mirror charges in a microfluidic chamber is a novel strategy to realize noninvasive action potential recording [169]. Nanodevices have shown potential, and we believe that scalable, low-cost and high-performance electrodes can be developed in the near future for theoretical investigation, clinical applications and commercialization. Second, with multiple functions indicating a new trend for in-cell nanoelectronics, in addition to intracellular recording, nanodevices are expected to append electrical/optical stimulation, biological/chemical sensing, intracellular delivery/extraction and other functions, which will facilitate the accurate regulation and synchronous monitoring of cells. Electrical stimulation is an effective strategy to regulate cell activity and expression, its combination with nanoelectronics is exciting for applications in biomedical environments, such as pacemakers for precise stimulation and synchronous monitoring. Microfluidic is a promising technology that can be combined with nanoelectronics to achieve multifunctional delivery and signal monitoring [170–172]. Finally, integrating nanodevices into implantable devices for intracellular recording in vivo is an exciting challenge. The reduced invasiveness of nanodevice to biological systems has the advantage of avoiding tissue inflammation and maintaining functional integrity in long-term connections. Based on a stable nanoelectronics-biology interface, it is possible to conduct large-scale studies of neuronal/cardiac circuit dynamics in vivo and even achieve the telemetric monitoring of physiological processes, as well as functional prosthetics of damaged neurons or cardiomyocytes. With future improvements, it is conceivable that the development of in-cell nanoelectronics will lead to significant breakthroughs in the research and clinical treatment of neuroscience, cardiology and associated pharmacology.
